# Multiparameter prediction model of immune checkpoint inhibitors combined with chemotherapy for non-small cell lung cancer based on support vector machine learning

**DOI:** 10.1038/s41598-023-31189-4

**Published:** 2023-03-18

**Authors:** Zihan Zhou, Wenjie Guo, Dingqi Liu, Jose Ramon Nsue Micha, Yue Song, Shuhua Han

**Affiliations:** 1 School of Medicine, Southeast University, Nanjing, 210009 China; 2 Department of Respiratory and Critical Care Medicine, Southeast University Zhongda Hospital, Nanjing, 210009 China; 3 College of Control Science and Engineering, Zhejiang University, Hangzhou, 310027 China; 4 Department of Radiology, BenQ Medical Center, Nanjing, 210000 China

**Keywords:** Medical research, Oncology, Risk factors

## Abstract

The reliable predictive markers to identify which patients with advanced non-small cell lung cancer tumors (NSCLC) will achieve durable clinical benefit (DCB) for chemo-immunotherapy are needed. In this retrospective study, we collected radiomics and clinical signatures from 94 patients with advanced NSCLC treated with anti-PD-1/PD-L1 combined with chemotherapy from January 1, 2018 to May 31, 2022. Radiomics variables were extracted from pretreatment CT and selected by Spearman correlation coefficients and clinical features by Logistics regression analysis. We performed effective diagnostic algorithms principal components analysis (PCA) and support vector machine (SVM) to develop an early classification model among DCB and non-durable benefit (NDB) groups. A total of 26 radiomics features and 6 clinical features were selected, and then principal component analysis was used to obtain 6 principal components for SVM building. RC-SVM achieved prediction accuracy with AUC of 0.91 (95% CI 0.87–0.94) in the training set, 0.73 (95% CI 0.61–0.85) in the cross-validation set, 0.84 (95% CI 0.80–0.89) in the external validation set. The new method of RC-SVM model based on radiomics-clinical signatures provides a significant additive value on response prediction in patients with NSCLC preceding chemo-immunotherapy.

## Introduction

Nowadays, for advanced non-small cell lung cancer tumors (NSCLC), immune checkpoint inhibitors (ICIs) combined with chemotherapy are recommended as standard first-line treatment without oncogenic driver alterations. However, a significant proportion of patients remain subjected to treatment resistance, efficacy biomarkers for ICIs plus chemotherapy are urgent needed to early forecast which patients will achieve durable clinical benefit (DCB)^1^.

The most established biomarker to predict the efficacy of immune checkpoint inhibitors (ICIs) in NSCLC is PD-L1 expression^2^. However, PD-L1 has some shortcomings including low sensitivity and specificity as a predictive biomarker of response^3,4^. Among the most recently recognized are ctDNA (circulating tumor DNA), and TMB (tumor mutational burden)^5,6^, but the testing technology of these biomarkers is immature and costly, and the conclusions of various researches are still inconsistent^7–9^. Therefore, routine tests of peripheral blood markers characterized by clinically convenient and practically noninvasive remain to be explored.

Radiomics applies data mining algorithms to medical images to obtain quantitative variables from tumor tissue imaging data^10,11^. Since tumors are heterogeneous in time and space^12^, it is possible to provide a more full-scale view of the tumor using imaging, which will make personalized treatment more accurate. As a noninvasive tool, radiomics can explore tumor heterogeneity, monitor tumor evolution, and evaluate treatment response. Therefore, the growing field of radiomic analysis has opened up new approaches to identify predictive biomarkers^13,14^. With the progress of computer science, machine learning has gradually applied to deal with large and complex data in medicine, which has been applied in many medical fields, including oncology, genomics, radiomics^15–17^. Support vector machines (SVM) is a new machine learning method based on statistical learning theory and high-dimensions groups have small sample sets can be classified accurately because multiple parameters in applied data sets has no negative effect on it^18^.

In this study, we developed baseline clinical information and radiomics signatures from pretreatment CT scans to predict response to immunotherapy combined with standard chemotherapy in patients with advanced NSCLC. We therefore set out to develop a multivariable model by SVM that integrated CT-based radiomics and clinical data for early identification of patients with advanced NSCLC who could realize durable clinical benefit from PD-(L)1 blockade-based ICI treatment and chemotherapy.

## Results

### Patient characteristics

A total of 94 patients were included in the study who accepted ICIs in combination with chemotherapy, including 20 patients for independent external validation. The clinical characteristics of these patients are summarized in Table 1. According to progression-free survival, 34 (45.9%) patients who did not achieve durable clinical benefit constituted the NDB group and the remaining 40 (54.1%) patients in the DCB group. Of the 94 patients, 4 without measurable lesions per RECIST 1.1, 90 had CT scans available for analysis of radiomics characteristics. 70 patients for training model and fivefold cross-validation, of which 38 (54.3%) were in the DCB group and 32 (45.7%) in the NDB group.

**Table 1:** Study population characteristics.

Parameter	Total n (%)	NDB group n (%)	DCB group n (%)
Total	74	34 (45.9%)	40 (54.1%)
Sex			
Male	61 (82.4%)	25 (41.0%)	36 (59.0%)
Female	13 (17.6%)	9 (69.2%)	4 (30.8%)
Age			
< 75 years	62 (83.8%)	29 (46.8%)	33 (53.2%)
≥ 75 years	12 (16.2%)	5 (41.7%)	7 (58.3%)
BMI			
< 18.5	4 (5.4%)	3 (75.0%)	1 (25.0%)
18.5–24	39 (52.7%)	16 (41.0%)	23 (59.0%)
≥ 24	26 (35.1%)	14 (53.8%)	12 (46.2%)
≥ 28	5 (6.8%)	1 (20.0%)	4 (80.0%)
Smoking state			
Never	21 (28.4%)	15 (71.4%)	6 (28.6%)
Former	30 (40.5%)	10 (33.3%)	20 (66.7%)
Current	23 (31.1%)	9 (39.1%)	14 (60.9%)
Histologic subtype			
Adenocarcinoma	49 (66.2%)	21 (42.9%)	28 (57.1%)
Squamous cell carcinoma	25 (33.8%)	13 (52.0%)	12 (48.0%)
Line of immunotherapy			
First	40 (54.1%)	16 (40.0%)	24 (60.0%)
Second	18 (24.3%)	9 (50.0%)	9 (50%)
≥ Third	16 (21.6%)	9 (56.3%)	7 (43.8%)
PD-L1 TPS, %			
< 1%	12 (16.2%)	9 (75.0%)	3 (25.0%)
1–50%	24 (32.4%)	13 (54.2%)	11 (45.8%)
≥ 50%	38 (51.4%)	12 (31.6%)	26 (68.4%)

### Univariable analysis of biomarkers for progression-free survival (PFS)

**Figure 1:**
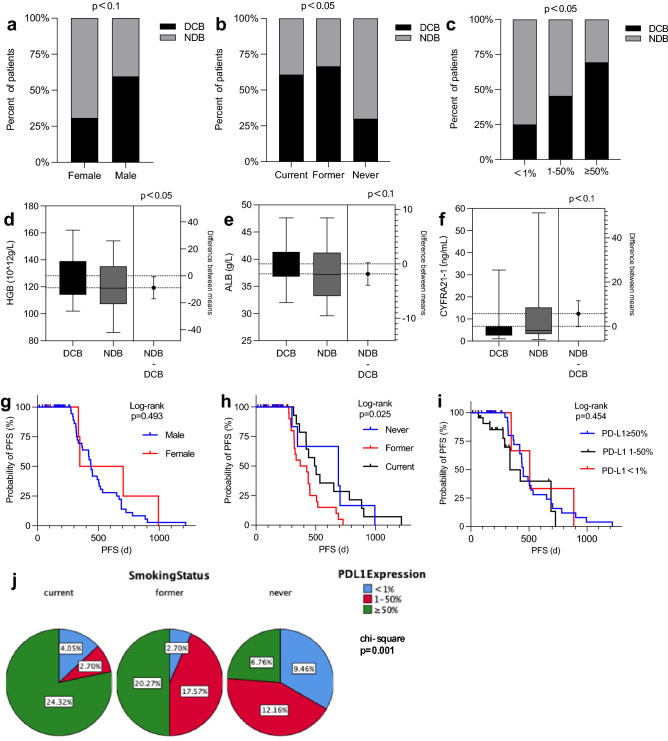
Pre-treatment clinical characteristics predict response to ICI plus chemotherapy. (**a**–**c**) Outcomes of patients stratified by Sex, Smoking status and PD-L1 expressing. p value was calculated by Univariate Logistic Regression Analysis (DCB n = 40; NDB n = 34). (**d**–**f**) Pre-treatment HGB, ALB and CYFRA21-1 in patients. p values were calculated using Univariate Logistic Regression Analysis. (**g**) Probability of PFS for male (median = 437 day) and female (median = 347 day). (**h**) Probability of PFS for current smokers (median = 689 day), former smokers (median = 336 day) and never smokers (median = 491 day). (**i**) Probability of PFS for PD-L1 expression < 1% (median = 507 day), PD-L1 expression ranged from 1 to 50% (median = 328 day) and PD-L1 expression ≥ 1% (median = 437 day). (**j**) Degree of smoking positively correlated with PD-L1 expression (p < 0.05).

We examined six basic characteristics and fourteen peripheral blood parameters measured before treatment initiation to identify clinical biomarker candidates for chemo-immunotherapy (Table 2). Univariable Logistics regression analysis of these factors revealed that males tended to be associated with better treatment benefits than females (p < 0.1). PD-L1 expression was identified as a significant predictor of durable clinical benefit (p < 0.05), notably, 3/12 (25%) patients with PD-L1 less than 1% also achieved DCB. Surprisingly, non-smokers seem to have a worse prognosis than smokers (p < 0.05). Among peripheral blood parameters, higher hemoglobin and albumin seem to tend to be associated with a better PFS (p < 0.05; p < 0.1). In contrast, a trend toward a worse PFS was apparent in patients with higher CFYRA-211 (p < 0.1). Furthermore, we observed that the degree of smoking appeared to be positively correlated with PD-L1 expression (p < 0.05). All the results are shown in Fig. 1.

**Table 2:** Univariate logistic regression analysis.

Parameter	Category	OR	95% CI	P Value
Sex	Male	3.24	0.898–11.695	0.073
Age	≥ 75 years	1.23	0.352–4.3	0.745
Histologic subtype	Adenocarcinoma	1.444	0.549–3.8	0.456
BMI	< 18.5			0.332
	18.5–23.9	4.312	0.411–45.282	0.223
	24–27.9	2.571	0.235–28.089	0.439
	> 28	12	0.514–280.089	0.122
Smoking status	Current	3.889	1.099–13.764	0.035
	Former	5	1.486–16.826	0.009
	Never			0.026
PD-L1	< 1%			0.026
	1–50%	2.538	0.548–11.766	0.234
	≥ 50%	6.5	1.487–28.407	0.013
WBC		1.048	0.953–1.153	0.334
HGB		1.035	1.005–1.066	0.023
ANC		1.039	0.957–1.128	0.356
ALC		0.955	0.831–1.098	0.517
AMC		2.207	0.438–11.13	0.337
dNLC		0.899	0.691–1.168	0.423
PLT		0.997	0.991–1.002	0.264
LDH		0.998	0.992–1.004	0.490
ALB		1.11	0.991–1.244	0.071
PNI		0.996	0.975–1.018	0.743
CEA		1.001	0.997–1.006	0.582
NSE		0.992	0.968–1.017	0.537
CYFRA21-1		0.96	0.918–1.004	0.072
ProGRP		1.003	0.978–1.028	0.827

### Radiomics features

Low reproducible features with an intraobserver ICC of 0.7 and less have been excluded, and twenty-six radiomics features with Spearman correlation coefficient of 0.3 or greater were retained which consist of 3 shape metrics (SM), texture features (TA) including 1 Gray Level Co-occurrence Matrix (GLCM), 1 Neighbouring Gray Tone Difference Matrix (NGTDM), 1 Gray Level Dependence Matrix (GLDM), wavelet features (WF) including 1 first order (FO), 1 NGTDM, 6 Gray Level Dependence Matrix (GLDM), 2 Gray Level Run Length Matrix (GLRLM), 4 Gray Level Run Length Matrix (GLRLM), 3 Neighbouring Gray Tone Difference Matrix (NGTDM), 2 Gray Level Size Zone Matrix (GLSZM), 1 Gray Level Co-occurrence Matrix (GLCM).

### Principal component analysis (PCA) and predictive model development

**Figure 2:**
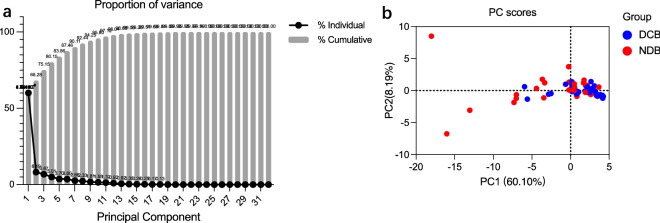
Principal component analysis of full feature signatures. (**a**) Figure of PCA value and cumulative variance contribution rate. Six main principal components were obtained from 32 features (including 6 clinical features and 26 radiomics features), and the cumulative variance contribution rate achieved 87.64%. (**b**) PCs scores of PC1 and PC2 in DCB group (blue dots) and NDB group (red dots).

After principal component analysis of the data, six main principal components were finally obtained from 32 features (including 6 clinical features and 26 radiomics features) by dimensionality reduction, and the cumulative variance contribution rate was 87.64% (Fig. 2a), which the six principal components could explain 87.64% of the total data, indicating that the principal components had a good interpretation effect on the whole. The first principal component (PC) extracted from all feature signatures primarily correlated with Gray Level Non-Uniformity, Surface Area and Busyness in radiomics (Proportion of variance = 60.3%). The first 2 PCs explained 68.28% of the total variance, which PC2 reflecting the Zone Variance and CTFRA21-1 (Supplemental Table S1). Figures 2b shows the two-dimensional scatter plots of the two PCs score between the DCB and NDB groups, which show differences between groups when we compared PCs. PC3 mainly contains characteristics of gender, smoking status, and PD-L1 expression. The last 3 PCs mostly reflect Correlation in Co-occurrence Matrix, Median in first-order wavelet features, ALB and HGB (Supplemental Table S2). Coefficient of components are showed in Supplemental Table S3.

**Figure 3:**
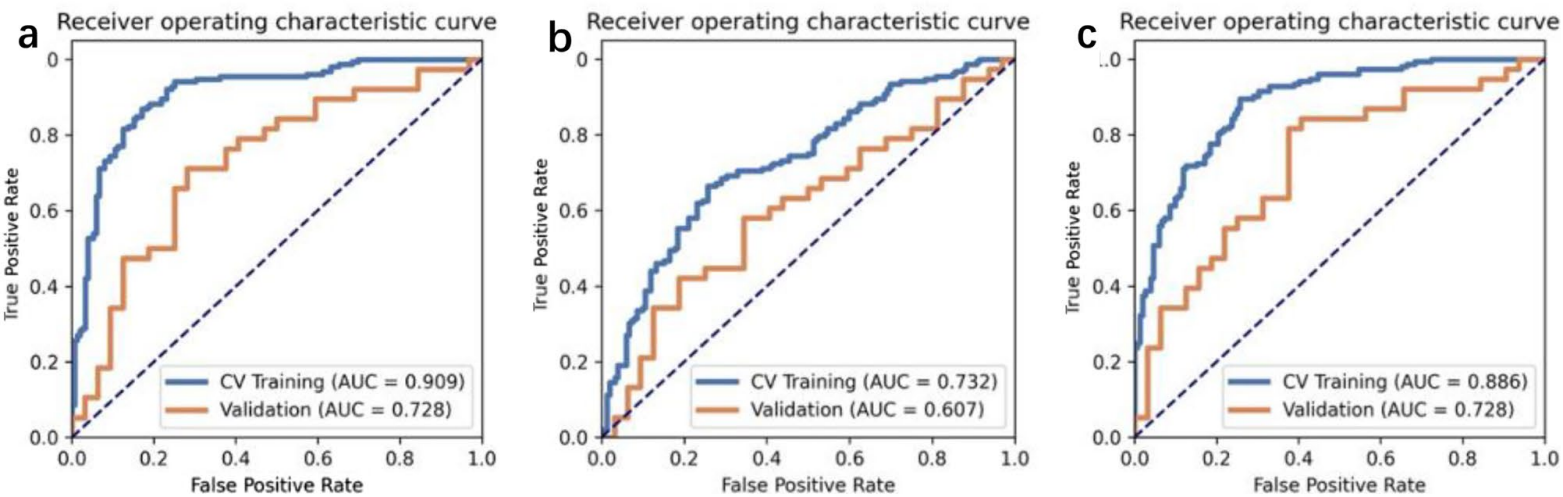
Receiver operating characteristic curves of (**a**) SVM, (**b**) LDA and (**c**) LR for predicting response to chemo-immunotherapy. *CV *cross validation, *AUC* area under the curve.

Regarding classification performance, RC (Radiomics-Clinical)-SVM training set achieved an AUC of 0.91 (95% CI 0.88, 0.94), the cross-validation set had an AUC of 0.73(95% CI 0.61, 0.85), and the external validation set had an AUC of 0.85(95% CI 0.80, 0.89). To compare the performance between the different models, we also tested the data by Linear Discriminant Analysis (LDA, AUC 0.73, CI 0.68, 0.79) and Logistic Regression (LR, AUC 0.89, CI 0.85, 0.92, Fig. 3). Clinical-only and radiomics-only signatures did not perform as well as the radiomics-clinical signature (Table. 3). Sensitivity, specificity, negative predictive value (NPV), and positive predictive value (PPV) for distinguishing patients with durable clinical benefit or not are summarized in Table 3.

**Table 3:** Radiomics-clinical, radiomics, and clinical-only signature performance in different machine learning models.

Models	AUC (95%CI)	Sensitivity (%)	Specificity (%)	PPV (%)	NPV (%)
Training set					
RC-SVM	0.91 (0.87–0.94)	86.8	82.9	83.5	86.3
RC-LDA	0.73 (0.68–0.79)	66.4	74.3	72.1	68.9
RC-LR	0.89 (0.85–0.92)	89.5	74.3	77.7	87.6
Cross-validation set					
RC-SVM	0.73 (0.61–0.85)	71.1	71.9	75.0	67.7
RC-LDA	0.61 (0.47–0.74)	57.9	65.6	66.7	56.8
RC-LR	0.73 (0.61–0.85)	81.6	62.5	72.1	74.1
External validation set					
RC-SVM	0.85(0.80–0.89)	86.6	60.0	86.6	60.0
Training set					
Rad-SVM	0.79 (0.69–0.85)	65.4	83.3	81.0	69.0
Cli-LR	0.84 (0.73–0.95)	76.9	66.7	73.2	71.0
Cross-validation set					
Rad-SVM	0.75 (0.67–0.83)	63.6	66.7	70.0	60.0
Cli-LR	0.84 (0.67–1.00)	91.7	62.6	57.1	93.2

*RC* Radiomics-Clinical, *SVM* Support Vector Machines, *LDA* Linear Discriminant Analysis, *LR* Logistic Regression, *Rad-SVM* Radiomics-SVM, *Cli-LR* Clinical-LR, *AUC* area under the curve, *NPV* negative predictive value, *PPV* positive predictive value.

Radiomics signature including 26 variables from histogram, shape, and local–regional texture.

Clinical signature including Sex, Smoking status, PD-L1 expressing, HGB, ALB and CYFRA21-1.

**Figure 4:**
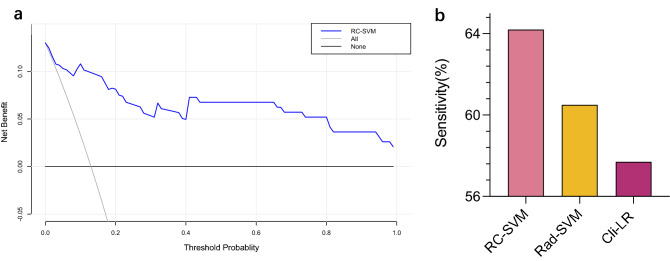
Clinical usefulness of radiomics-clinical SVM. *RC* Radiomics-Clinical, *SVM* Support Vector Machines, *LDA* Linear Discriminant Analysis, *LR* Logistic Regression, *Rad-SVM* Radiomics-SVM, *Cli-LR* Clinical-LR. (**a**) Decision curve analysis of RC-SVM, with a threshold range of about 0.12–0.98 and a maximum net benefit of about 0.13. (**b**) Sensitivity at 95% specificity for the RC-SVM, Rad-SVM and Cli-LR (64.2%, 60.5% and 57.7%).

**Figure 5:**
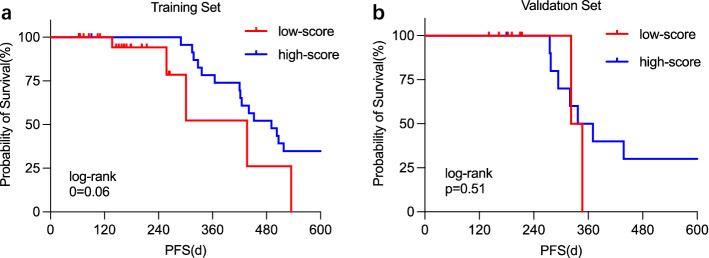
Kaplan–Meier overall survival curve analysis in training (**a**) and validation set (**b**). (**a**) Probability of PFS for high-score (median = 493 day) and low-score (median = 297 day) in training set. (**b**) Probability of PFS for high-score (median = 347 day) and low-score (median = 337 day) in validation set. The CT-based radiomics and clinical score was defined as high (> 0) or low (≤ 0), score = Features * β Model Coefficient + Bias Model Coefficient.

Next, we estimated clinical usefulness by decision curve analysis and counting the sensitivity at 95% specificity. The decision curve analysis provided insight into the range of predicted risks, and results showed that the model provided benefit value to patients, with a threshold range of about 0.12–0.98 and a maximum net benefit of about 0.13 (Fig. 4a). The sensitivity for the Radiomics-Clinical-SVM, Radiomics-SVM, and Clinical-LR was 64.2% (95% CI 59.7 to 68.3%), 60.5% (95% CI 56.2 to 64.3%), and 57.7% (95% CI 53.8 to 61.5%) at 95% specificity (Fig. 4b). Overall performance of the RC-SVM was quantified as the Brier score, we obtained that the Brier scores of the training set and the cross-validation set were 0.1 and 0.17, which were both close to 0, indicating good prediction ability. Furthermore, patients with a high score (score > 0) calculated by radiomics and clinical features showed improved PFS compared with those with a low score (score ≤ 0), but this difference was not significant (p = 0.06 in training set, p = 0.0.51 in validation set, Fig. 5).

## Discussion

Although immune checkpoint inhibitors have shown conspicuous promise for the treatment of advanced NSCLC, response rates remain suboptimal, and reliable predictive markers to identify which patients will achieve durable clinical benefit for chemo-immunotherapy are needed. In addition, most of the existing studies aimed to develop prediction model for mono-immunotherapy, as far as we know, a significant part of patients received ICIs combined with chemotherapy as first-line therapy to avoid hyperprogression. To our knowledge, few studies have published which develop principal component analysis and support vector machines based on a comprehensive analysis of a wide range of baseline pretreatment clinicopathological factors and radiomic features that may accurately predict clinical prognosis for chemo-immunotherapy in NSCLC. Unlike many proposed molecular markers under development (e.g. ctDNA and TMB), these conventional clinical parameters and CT scans are readily available in the clinic at the start of treatment. They should be useful tools when discussing first or further lines of treatment with patients.

Our study suggests that CT-based radiomics features can be used to distinguish the prognosis of the NSCLC patients with immunochemotherapy. Imaging reflects features of tumors, including PD-L1 expression status and CD8 infiltration^11,19^. Imaging features have been proved are related to genomic profiles which called radiogenomics and it is to applied in assessment of the response to immunotherapy in patients with non-small cell lung cancer and glioblastomas^20,21^.

In this study, we developed principal component analysis to reduce the dimension of radiomics features and retained features with diagnostic value that decrease the effects of redundant features on model classification. Most of the features included in the model are wavelet-transformed second-order texture features, and only 5 are the original first-order texture features. The second-order texture features can describe the variation of gray level between adjacent voxels, while the wavelet transform filters from original features. The results of this study suggest that the wavelet transformed second-order texture may be more valuable in reflecting the microscopic information of tumors. Furthermore, we observed that the negative coefficient of Gray Level non-uniformity, Busyness, and Zone Variance features, maybe indicating homogeneity of tumors, were associated with a better immune response. Conversely, radiomics features reflecting lesion size like Surface Area were associated with a worse probability of response.

We performed SVM to establish the classification model, and applied two other machine learning algorithms including logistic regression and linear discriminant analysis, to compare the classification accuracy and evaluate the model performance by calculating the area under the curve (AUC), accuracy, sensitivity, PPV and NPV. RC-SVM achieved good prediction accuracy and clinical usefulness in the training set with an AUC of 0.91, a 86.8% sensitivity and a specificity of 82.9%. It performs well in the cross-validation set and external validation set, and the other indicators were also higher than LR and LDA. In RC-SVM, patients with higher scores were also shown to have longer PFS and better prognostic outcomes.

Moreover, we suppose there is a plausible biological explanation for clinical components of our model: (1) Smoking status: it may be linked to the number of tumor copies, which, in turn, increases immunogenicity, resulting in better response to ICI at higher levels^22–24^; (2) PD-L1 expression: a factor in the tumor microenvironment which can act to modulate the existing activated antitumor T cell immune response is an approved biomarker to predict PD-(L)1 blockade in NSCLC^25^; (3) Hemoglobin: In our study, higher hemoglobin is associated with longer PFS with chemo-immunotherapy, however, the biology behind this finding is yet to be defined and few studies have explored hemoglobin's role as a biomarker for predicting immunotherapy outcomes. Tumor hypoxia, a characteristic of the immunosuppressive tumor microenvironment^26^, has been proven to cause resistance to current anticancer therapeutics including chemotherapy, photodynamic therapy, radiotherapy and immunotherapy^27,28^. Therefore, high level of hemoglobin, might indicating an oxygen-enriched environment which is characterized by having higher nutrient availability and by being rather immunostimulatory, was associated with a more efficient treatment response. (4) CFYRA21-1: serum level of CFYRA21-1 is related to the tumor size, lymph node status, distant Metastasis and the stage of disease^29,30^ which could assist in predicting immunotherapy efficacy^31^ and baseline level was positively correlated with the patient’s risk score before immunotherapy^32^.

Our study had some limitations. First, due to the retrospective, there could not be standardization of image acquisition parameters. Second, the sample size is insufficient according to the PROBAST guidelines. To solve this dilemma, we will keep collecting NSCLC patients receiving immunotherapy combined with chemotherapy on the one hand, and explore new methods of small sample learning to optimize the model on the other hand. Third, in some cases, patients received ICIs as a secondary line of treatment or beyond, which may have had an impact on their nutritional status and baseline inflammation, although the number of lines of immunotherapy was not related to clinical outcomes of our study. In addition, since some patients did not routinely receive next-generation sequencing during the sample collection period, the targetable driver alterations were not counted in this study. Thus, further exploration is necessary to determine how the model would perform in these different patient subsets.

In summary, our model which has the advantages of non-invasiveness, convenience, and economy integrated multidimensional data including routinely collected clinical factors and CT-based radiomics signature. Ultimately, it is expected to provide new tools for early prognostic assessment and guiding treatment decisions for NSCLC, thereby promoting individualized treatment of lung cancer, possibly contributing to the selection of the most appropriate ICI treatment for patients with NSCLC, reducing the treatment cost of patients and improve their survival and quality of life.

## Method

### Study population and therapy scheme

**Figure 6:**
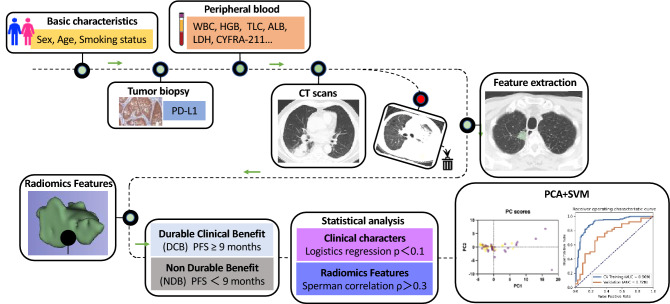
Study flowchart.

From January 1, 2018 to May 31, 2022, we consecutively included 94 patients with the initial diagnosis of NSCLC, for whom a whole-body assessment revealed tumor stage IIIB to IV or recurrent NSCLC and who were subsequently treated with anti-PD-1/PD-L1 combination chemotherapy at Zhongda Hospital Southeast University, of which 20 patients did not participate in model training as independent external validation. This study excluded participants who met the following criteria: patients with missing follow-op data, who had complicated factors such as infectious fever, blood system or immune system diseases that may affect blood test results, discontinuation of the drug due to intolerable toxic effects (non-disease progression), and non-measurable lesions as per Response Evaluation Criteria in Solid Tumors (RECIST) 1.1. The study was approved by the ethics review board at Zhongda Hospital Southeast University with a reference number of No.2021ZDSYLL213-P01 and was performed by the Declaration of Helsinki. Informed consent was obtained from all participants. As shown in Fig. 6, the study flowchart illustrates the process.

### Image acquisition and analysis

We obtained and analyzed CT scans within 30 days before the date of treatment start. CT scans were acquired by using 64-channel CT scanners (SIEMENS SOMATOM) in the axial plane with tube voltage of 120 kV, and section interval of 1.25–5 mm. All lung lesions for each patient were segmented twice by a physician, blinded to the clinical outcome, with 5 years of experience in oncologic imaging, by using the 3DSlicer version 5.1.9 (3D Slicer, National Institutes of Health, Bethesda, MD, USA) semiautomatic contouring tool. Radiomics features based CT (first order, shape, texture and wavelet) were generated by using the SlicerRadiomics package (version a57d142) for Python (version 3.7.1). The following features classes were extracted: statistical first order (FO), shape metrics (SM), texture features (TA) including Gray Level Co-occurrence Matrix (GLCM), Gray Level Run Length Matrix (GLRLM), Gray Level Size Zone Matrix (GLSZM), Neighbouring Gray Tone Difference Matrix (NGTDM), Gray Level Dependence Matrix (GLDM), wavelet features (WF) for all the above features, except shape, were calculated by combining High and Low pass filters for each dimension. In total, we extracted 851 features from each image volume (18 FO, 14SM, 24 GLCM, 16 GLRLM, 16 GLSZM, 14 GLDM, 5 NGTDM, and 744 WF).

### Clinical data

Data from electronic records was used to collect basic patient information and laboratory results. These included age, sex, tumor type, BMI, Smoking status, PD-L1, white blood cell count (WBC), red blood cell count (RBC), absolute lymphocyte count (ALC), absolute monocyte count (AMC), derive neutrophil-to-lymphocyte ratio (dNLR), platelet count (PLT), lactate dehydrogenase (LDH), albumin level (ALB), prognostic nutritional index (PNI, ALB + ALC × 5), carcinoembryonic antigen (CEA), neuron-specific enolase (NSE), pro-gastrin-releasing peptide (ProGRP), and CYFRA21-1. Outcome data, similar to the concept of durable clinical benefits (DCB) proposed by previous studies^33^, defined DCB as PFS of at least 9 months from ICIs-based combination therapy and non-durable benefit as PFS < 9 months from combination treatment.

### Statistical analysis and model building

PCA was applied in our algorithm, it is a multivariate statistical technique that turns a series of initial relevant variables to fresh irrelevant variables named principal components according to the maximum variance. According to their similar variances, PCA can be used to classify. The PC scores show the intensity of each PC loading in dataset group and the first PC loading shows the most corrected clinical benefit features in the dataset. Then we could identify durable clinical benefit or not through the scores from individuals of a population according to variations of the clinical and radiomics characteristics^34^.

In the classification part of algorithm we perform SVM which is a technology used for binary classification and the basic principle is to render a problem linearly separable by making use of a nonlinear mapping function that transforms data in input space to data in feature space^35^. The SVM will discovers the optimal separating hyperplane automatically and achieve accurate classification despite small sample sets and multiple parameters. At the same time, we use regularization in support vector machine to avoid overfitting problem. AUC, accuracy, sensitivity, positive predictive value and negative predictive value were calculated by PCA and SVM analysis of the whole dataset collected from the patients by using the Statistics Toolbox of MATLAB R2021b (Math Works, Natick, MA). Brier score was calculated based on the prediction probability calculated by the model in MATLAB. The calculation formula of Brier score is BS = $\frac{1}{N}\sum_{t=1}^{N}{\left({f}_{t}-{o}_{t}\right)}^{2}$. The DCA curve was performed using R software (4.2.2).

SPSS (version r2021b) was used for statistical analysis. Logistics regression analysis was applied to find independent clinical indicators associated with clinical benefit. We incorporated the statistically significant factors in the univariate analysis into the PCA. A p value < 0.1 was considered statistically significant. Spearman correlation coefficients were calculated for radiomics features, and we incorporated elements with correlation coefficients greater than 0.3 into the PCA. A fivefold cross-validation analysis internally validated model predictions.

## Supplementary Information

Supplementary Tables.

## Data Availability

The datasets generated and analysed during the current study are available from the corresponding author on reasonable request.
