# Supplementary material for: Multiparameter prediction model of immune checkpoint inhibitors combined with chemotherapy for non-small cell lung cancer based on support vector machine learning
Source: Sci Rep. 2023 Mar 18;13:4469. doi: 10.1038/s41598-023-31189-4 (PMC10024716; doi:10.1038/s41598-023-31189-4)
Supplement: Supplementary file 1 — Supplementary Tables. [file 41598_2023_31189_MOESM1_ESM.docx]

**Multiparameter Prediction Model of Immune Checkpoint Inhibitors Combined with Chemotherapy for Non-small Cell Lung Cancer Based on Support Vector Machine Learning**

Zihan Zhou^1,2^, Wenjie Guo^3^, Dingqi Liu^4^, Jose Ramon Nsue Micha^1,2^, Yue Song^1,2^, Shuhua Han^1,2*^

^1^ School of Medicine, Southeast University, Nanjing 210009, China.

^2^ Department of Respiratory Medicine, Southeast University Zhongda Hospital, Nanjing 210009, China.

^3^ College of Control Science and Engineering, Zhejiang University 310027, Hangzhou, China

^4^ Department of Radiology, BenQ Medical Center, Nanjing 210000, China

**^*^** Corresponding author: Email: [hanshuhua0922@126.com](mailto:hanshuhua0922@126.com)

| **Total Variance Explanation** | | | | | | |
| --- | --- | --- | --- | --- | --- | --- |
| Component | Initial Eigenvalue | | | Extract the Sum of Squared | | |
|  | Total | Persentage of Variance | Cumulative Variance | Total | Persentage of Variance | Cumulative Variance |
| 1 | 19.231 | 60.096 | 60.096 | 19.231 | 60.096 | 60.096 |
| 2 | 2.620 | 8.187 | 68.283 | 2.620 | 8.187 | 68.283 |
| 3 | 2.197 | 6.865 | 75.149 | 2.197 | 6.865 | 75.149 |
| 4 | 1.602 | 5.006 | 80.154 | 1.602 | 5.006 | 80.154 |
| 5 | 1.185 | 3.702 | 83.856 | 1.185 | 3.702 | 83.856 |
| 6 | 1.152 | 3.599 | 87.456 | 1.152 | 3.599 | 87.456 |
| 7 | .851 | 2.658 | 90.114 |  |  |  |
| 8 | .745 | 2.329 | 92.443 |  |  |  |
| 9 | .578 | 1.806 | 94.249 |  |  |  |
| 10 | .495 | 1.546 | 95.796 |  |  |  |
| 11 | .423 | 1.322 | 97.117 |  |  |  |
| 12 | .295 | .923 | 98.041 |  |  |  |
| 13 | .197 | .615 | 98.656 |  |  |  |
| 14 | .111 | .347 | 99.003 |  |  |  |
| 15 | .082 | .258 | 99.260 |  |  |  |
| 16 | .080 | .251 | 99.512 |  |  |  |
| 17 | .054 | .168 | 99.680 |  |  |  |
| 18 | .040 | .126 | 99.806 |  |  |  |
| 19 | .028 | .087 | 99.894 |  |  |  |
| 20 | .012 | .038 | 99.932 |  |  |  |
| 21 | .007 | .021 | 99.953 |  |  |  |
| 22 | .007 | .020 | 99.974 |  |  |  |
| 23 | .005 | .015 | 99.989 |  |  |  |
| 24 | .003 | .010 | 99.998 |  |  |  |
| 25 | .000 | .001 | 99.999 |  |  |  |
| 26 | .000 | .000 | 100.000 |  |  |  |
| 27 | 5.435E-5 | .000 | 100.000 |  |  |  |
| 28 | 4.275E-5 | .000 | 100.000 |  |  |  |
| 29 | 1.334E-5 | 4.170E-5 | 100.000 |  |  |  |
| 30 | 8.162E-6 | 2.551E-5 | 100.000 |  |  |  |
| 31 | 3.847E-6 | 1.202E-5 | 100.000 |  |  |  |
| 32 | 1.441E-8 | 4.502E-8 | 100.000 |  |  |  |

**Supplementary Table S1. Total variance explanation of PCA.** The cumulative variance contribution rate **of s**ix main principal components obtained from 32 features achieved 87.64% which means six principal components could explain 87.64% of the total data.

| **Component Matrix** | | | | | | |
| --- | --- | --- | --- | --- | --- | --- |
|  | component | | | | | |
|  | 1 | 2 | 3 | 4 | 5 | 6 |
| Sex | -.053 | .042 | .678 | .247 | .189 | -.097 |
| Smokingstatus | .044 | -.174 | -.639 | -.158 | -.363 | .203 |
| PDL1expression | -.147 | .005 | .675 | .015 | .150 | .306 |
| HGB | -.371 | .311 | .086 | .020 | .171 | -.633 |
| ALB | -.265 | .443 | .031 | .040 | .439 | -.219 |
| CYFRA211 | .517 | -.656 | .339 | -.028 | -.102 | .047 |
| ori_shape_LeastAxisLength | .649 | -.404 | -.296 | -.046 | .304 | -.272 |
| ori_shape_MinorAxisLength | .659 | -.350 | -.423 | -.056 | .297 | -.161 |
| ori_shape_SurfaceArea | .889 | -.337 | -.022 | -.089 | .134 | -.098 |
| ori_GLCM_Correlation | .462 | .077 | -.168 | .821 | -.007 | .141 |
| ori_GLDM_GrayLevelNonUniformity | .959 | .078 | .077 | -.127 | -.036 | .018 |
| ori_NGTDM_Busyness | .863 | -.400 | .203 | -.016 | -.028 | -.068 |
| wavelet_LLH_FO_Median | .369 | -.141 | -.449 | -.018 | .559 | .220 |
| wavelet_LLH_NGTDM_Busyness | .878 | -.154 | .118 | .083 | -.124 | -.045 |
| wavelet_LHL_GLDM_GrayLevelNonUniformity | .966 | .228 | .035 | -.024 | -.027 | .033 |
| wavelet_LHL_GLRLM_GrayLevelNonUniformity | .988 | -.001 | .106 | -.051 | -.023 | .005 |
| wavelet_LHH_GLDM_GrayLevelNonUniformity | .974 | .203 | .042 | -.019 | -.011 | .026 |
| wavelet_LHH_GLRLM_GrayLevelNonUniformity | .988 | -.009 | .111 | -.035 | -.012 | .003 |
| wavelet_LHH_NGTDM_Strength | -.328 | .035 | .152 | -.426 | .441 | .541 |
| wavelet_HLL_GLDM_GrayLevelNonUniformity | .972 | .179 | -.002 | -.092 | -.010 | .009 |
| wavelet_HLL_GLRLM_GrayLevelNonUniformity | .983 | -.047 | .085 | -.094 | -.014 | -.011 |
| wavelet_HLL_GLSZM_LargeAreaEmphasis | .758 | .582 | -.174 | -.096 | -.041 | .034 |
| wavelet_HLL_GLSZM_ZoneVariance | .758 | .582 | -.174 | -.096 | -.041 | .034 |
| wavelet_HLL_NGTDM_Busyness | .762 | .383 | .069 | .171 | -.062 | .113 |
| wavelet_HLH_GLDM_GrayLevelNonUniformity | .978 | .144 | .021 | -.067 | .006 | .005 |
| wavelet_HLH_GLRLM_GrayLevelNonUniformity | .983 | -.063 | .096 | -.069 | -.001 | -.016 |
| wavelet_HHL_GLDM_GrayLevelNonUniformity | .965 | .231 | -.009 | -.016 | .010 | .025 |
| wavelet_HHL_GLRLM_GrayLevelNonUniformity | .992 | .049 | .053 | -.029 | .006 | .003 |
| wavelet_HHH_GLDM_GrayLevelNonUniformity | .975 | .175 | .010 | -.013 | .023 | .016 |
| wavelet_HHH_GLRLM_GrayLevelNonUniformity | .991 | -.004 | .071 | -.022 | .017 | -.006 |
| wavelet_LLL_GLCM_Correlation | .494 | -.189 | -.157 | .730 | .158 | .196 |
| wavelet_LLL_NGTDM_Busyness | .838 | -.387 | .163 | -.088 | -.067 | -.150 |

**Supplementary Table S2. Component Matrix of PCA.** The first principal component (PC) primarily is correlated with Gray Level Non-Uniformity, Surface Area and Busyness in radiomics. The first 2 PCs primarily reflects the Zone Variance and CTFRA21-1. PC3 mainly contains characteristics of gender, smoking status, and PDL1 expression. The last 3 PCs mostly reflect Correlation in Co-occurrence Matrix, Median in first-order wavelet features, ALB and HGB.

**Supplementary Table S3. Component Score Coefficient Proof of PCA.**

| **Component Score Coefficient Proof** | | | | | | |
| --- | --- | --- | --- | --- | --- | --- |
|  | Component | | | | | |
|  | 1 | 2 | 3 | 4 | 5 | 6 |
| Sex | -.003 | .016 | .309 | .154 | .159 | -.084 |
| Smokingstatus | .002 | -.067 | -.291 | -.099 | -.307 | .176 |
| PDL1expression | -.008 | .002 | .307 | .010 | .127 | .266 |
| HGB | -.019 | .119 | .039 | .012 | .144 | -.549 |
| ALB | -.014 | .169 | .014 | .025 | .370 | -.190 |
| CYFRA211 | .027 | -.250 | .154 | -.018 | -.086 | .041 |
| ori_shape_LeastAxisLength | .034 | -.154 | -.135 | -.029 | .257 | -.236 |
| ori_shape_MinorAxisLength | .034 | -.134 | -.193 | -.035 | .251 | -.140 |
| ori_shape_SurfaceArea | .046 | -.129 | -.010 | -.056 | .113 | -.086 |
| ori_GLCM_Correlation | .024 | .030 | -.077 | .513 | -.006 | .122 |
| ori_GLDM_GrayLevelNonUniformity | .050 | .030 | .035 | -.079 | -.030 | .016 |
| ori_NGTDM_Busyness | .045 | -.153 | .093 | -.010 | -.024 | -.059 |
| wavelet_LLH_FO_Median | .019 | -.054 | -.205 | -.012 | .472 | .191 |
| wavelet_LLH_NGTDM_Busyness | .046 | -.059 | .054 | .052 | -.104 | -.039 |
| wavelet_LHL_GLDM_GrayLevelNonUniformity | .050 | .087 | .016 | -.015 | -.023 | .029 |
| wavelet_LHL_GLRLM_GrayLevelNonUniformity | .051 | -.001 | .048 | -.032 | -.020 | .004 |
| wavelet_LHH_GLDM_GrayLevelNonUniformity | .051 | .077 | .019 | -.012 | -.010 | .022 |
| wavelet_LHH_GLRLM_GrayLevelNonUniformity | .051 | -.003 | .050 | -.022 | -.010 | .002 |
| wavelet_LHH_NGTDM_Strength | -.017 | .013 | .069 | -.266 | .373 | .470 |
| wavelet_HLL_GLDM_GrayLevelNonUniformity | .051 | .068 | -.001 | -.057 | -.009 | .008 |
| wavelet_HLL_GLRLM_GrayLevelNonUniformity | .051 | -.018 | .039 | -.059 | -.011 | -.010 |
| wavelet_HLL_GLSZM_LargeAreaEmphasis | .039 | .222 | -.079 | -.060 | -.035 | .029 |
| wavelet_HLL_GLSZM_ZoneVariance | .039 | .222 | -.079 | -.060 | -.035 | .029 |
| wavelet_HLL_NGTDM_Busyness | .040 | .146 | .032 | .107 | -.053 | .098 |
| wavelet_HLH_GLDM_GrayLevelNonUniformity | .051 | .055 | .009 | -.042 | .005 | .004 |
| wavelet_HLH_GLRLM_GrayLevelNonUniformity | .051 | -.024 | .044 | -.043 | .000 | -.014 |
| wavelet_HHL_GLDM_GrayLevelNonUniformity | .050 | .088 | -.004 | -.010 | .009 | .022 |
| wavelet_HHL_GLRLM_GrayLevelNonUniformity | .052 | .019 | .024 | -.018 | .005 | .002 |
| wavelet_HHH_GLDM_GrayLevelNonUniformity | .051 | .067 | .005 | -.008 | .020 | .013 |
| wavelet_HHH_GLRLM_GrayLevelNonUniformity | .052 | -.001 | .032 | -.014 | .014 | -.005 |
| wavelet_LLL_GLCM_Correlation | .026 | -.072 | -.072 | .455 | .133 | .170 |
| wavelet_LLL_NGTDM_Busyness | .044 | -.148 | .074 | -.055 | -.057 | -.130 |
